# Development of a public health emergency preparedness competency model for European Union countries

**DOI:** 10.2807/1560-7917.ES.2018.23.48.1700631

**Published:** 2018-12-06

**Authors:** Michael A. Stoto, Elena Savoia, Christopher Nelson, Rachael Piltch-Loeb, Stefano Guicciardi, Judit Takacs, Carmen Varela Santos, Massimo Ciotti

**Affiliations:** 1 Center for Global Health Science and Security, Georgetown University, Washington, United States; 2 Emergency Preparedness Research, Evaluation & Practice Program, Harvard T.H. Chan School of Public Health, Boston, United States; 3 RAND Corporation, Santa Monica, United States; 4 College of Global Public Health, New York University, New York, United States; 5 Department of Biomedical and Neuromotor Sciences, University of Bologna, Bologna, Italy; 6 Public Health Capacity and Communication Unit, European Centre for Disease Prevention and Control, Stockholm, Sweden

**Keywords:** public health emergency preparedness, competency model, detection and assessment, surveillance, policy development, adaptation and implementation, emergency risk communication

## Abstract

In 2017, the European Centre for Disease Prevention and Control (ECDC) developed a competency model for individuals who work in public health emergency preparedness (PHEP) in European Union (EU) countries. The model serves as the basis for developing competency-based training programmes to support professionals in PHEP efforts at the country level. The competency model describes the knowledge and skills professionals need when working in national-level PHEP, such as preparedness committee members or their equivalents. In order to develop the model, existing competency statements were reviewed, as well as case studies and reports. Fifty-three professionals from the EU and other countries provided feedback to the model by participating in a three-stage consultation process. The model includes 102 competency, 100 knowledge and 158 skill statements. In addition to specifying the appropriate content for training programmes, the proposed common competency model can help to standardise terminology and approaches to PHEP training.

## Background

Pursuant to Decision 1082 of the European Parliament and Council on serious cross-border threats to health [1], the European Centre for Disease Prevention and Control (ECDC) has worked to identify strengths and areas for improvement of public health emergency preparedness (PHEP) in the European Union (EU). This work began with the development of a PHEP logic model focusing on cross-border threats to health in the European context [2]. This logic model served as basis for developing the competency-based model described in this manuscript. The PHEP logic model provides both a framework for assessing preparedness and, in its list of PHEP capabilities, a language for identifying gaps in preparedness. The logic model distinguishes between capacities and capabilities. In line with our earlier work, capacities represent the resources—infrastructure, policies and procedures, knowledgeable and trained personnel—that a public health system has to draw upon. Capabilities describe what countries are expected to achieve during an emergency [2].

The PHEP logic model includes a list of capabilities in five preparedness areas: (i) detection and assessment (incident recognition, risk characterisation, surveillance and epidemiological monitoring, laboratory analysis, environmental monitoring); (ii) policy development, adaptation and implementation (policy development and adaptation for infection control and treatment guidance, policy development and adaptation for population-based disease control, policy implementation for communicating between national and subnational authorities and enforcing laws and regulations); (iii) health services (preventive services; medical surge; management of medical countermeasures, supplies and equipment; medical services for healthcare workers and emergency responders); (iv) coordination and communication (crisis management; communication with healthcare providers; communication with emergency management, public safety and other sectors; communication with other public health agencies at the global, European, national and subnational levels); (v) emergency risk communication with the general public (addressing communication inequalities; generating dynamic listening and managing rumours; communicating risk in an accurate, transparent and timely manner; generating and maintaining trust) [2].

The capabilities in the PHEP logic model are system-level characteristics. To take this work further, we linked these capabilities to the competencies professionals should achieve to effectively prepare for and respond to public health emergencies. The Association of Schools of Public Health in the European Region (ASPHER) has taken a similar approach in developing individual competencies [3] related to system capabilities, i.e. in their Essential Public Health Operations (EPHOs) developed for the World Health Organization Regional Office for Europe (WHO/Europe) [4].

Here we describe the development of the ECDC competency model for PHEP [5] and potential target audiences for the related training. We also summarise the consultation process used to identify specific competencies and illustrate the results by referring to a specific capability as an example.

### Development of workforce-specific public health emergency preparedness competencies

Competency-based trainings are characterised by two features: (i) learning outcomes, i.e. the required competencies are precisely defined, so as to be measurable, (ii) preparation for specific jobs or professional roles, from which the competencies are derived. Such trainings are typically implemented in a modular format based on level of difficulty and/or specificity. This competency model was created to facilitate the standardisation of preparedness education and training across various public health systems. The model is designed to accommodate differences among preparedness systems, unique response demands, infrastructural levels, vulnerabilities of specific public health systems and differences in threats, and should be tailored specifically to individuals’ developmental trajectories.

Competencies are combinations of knowledge and skills that a professional is required to have in order to perform a task effectively. In the proposed model, we used the definitions of knowledge and skills based on the European Qualifications Framework (EQF) for lifelong learning. Knowledge is the outcome of the assimilation of information – facts, principles, theories and practices – through learning. Skills, on the other hand, are the ability to apply knowledge and use know-how to complete tasks and solve problems. Skills can be cognitive (involving the use of logical, intuitive and creative thinking) or practical (involving the use of methods, materials, tools and instruments) [6]. Competencies are often described as ‘KSAs’, with K and S standing for ‘knowledge’ and ‘skills’, respectively. The A sometimes stands for ‘attitudes’ and other times for ‘abilities’, neither of which are consistently defined, so we chose to focus on knowledge and skills only, incorporating abilities into the latter category.

The target audience of the PHEP competencies presented in the model are members of national preparedness committees or similar bodies in Europe. However, PHEP systems vary markedly among EU countries, reflecting different governmental structures, resources and histories. As a result, the profile of individuals with primary responsibility in the many functional areas related to PHEP varies. Depending on the country, different functional areas might be executed under different organisational structures. For example, in some countries there may be one responsible official for multiple functions, whereas in other countries some functional areas might not be explicitly represented at all. In addition, the professionals engaged in any particular emergency response will depend on the nature of the event. Thus, in the proposed model we did not identify a single set of core competencies, but rather considered a wide range of workforce categories (e.g. epidemiologists, policy makers, risk communicators). Unlike other ECDC competency statements that are targeted at experts with specific responsibilities in epidemiology [7], microbiology [8] or vaccine-preventable diseases [9], the PHEP competencies presented in this model are intended for professionals working in coordinating roles in PHEP at the national level. Consequently, the competencies cover a broad range of topics. Countries will have to decide how to apply the competency model to ensure that, as a group, members of national preparedness committees or their equivalents possess the listed required knowledge and skills to effectively respond to public health emergencies.

### Development of knowledge and skill statements

A team of experts in PHEP assessment and training developed the knowledge and skill statements. The first step in developing the model consisted of drafting a preliminary list of PHEP competencies, drawing on three sources of information: (i) case studies on specific public health emergencies, in order to identify PHEP system capabilities that were called upon during the response; (ii) existing competency statements [7-9] and (iii) a review of the literature on PHEP and training and evaluation guidance tools developed by the World Health Organization (WHO) [10-13]. The result of this process was a list of competencies and knowledge and skill statements grouped into five categories defined by related system-level capability.

We then conducted a three-stage consultation process. In the first stage, we presented a draft of the competencies, knowledge and skill statements at the joint meeting of the ECDC coordination committees for preparedness, communication and public health training in September 2016 in Stockholm, Sweden. Representatives of 12 EU/European Economic Area (EEA) countries participated in the discussion and provided feedback on the draft.

For the second stage, we consulted with a group of practitioners with experience in emergency preparedness and response who volunteered to complete an online modified DELPHI process [5]. Additionally, invitation leaflets were distributed among the European Scientific Conference on Applied Infectious Disease Epidemiology (ESCAIDE) 2016 conference participants. Participants in the consultation process were presented with the draft list of competencies, knowledge and skill statements, and were asked whether they agreed or disagreed with the inclusion of the statements and their wordings. They were invited to comment on and revise the document, as well as to make suggestions about additional knowledge or skills they thought should be included. A total of 28 individuals participated, including representatives from international organisations, ministries and departments of health, academia, and other organisations. Overall, the participants worked in 17 EU/EEA countries and 32 other countries.

In the third stage of the consultation process, we sent a draft of the competency model and related report—including the revised list of competencies, knowledge and skill statements—to some of the most active participants in the second stage of the consultation and the ECDC National Focal Points both for Preparedness and Response and for Public Health Training for additional review.

Considering the three stages together, input from 53 individuals from EU and other countries was incorporated. The participants’ professional areas of expertise included medicine and public health, epidemiology and surveillance, microbiology, crisis management, communication, emergency preparedness, health policy and law.

## Consultation process outcome

The process resulted in a list of 102 competency and 100 knowledge and 158 skill statements covering the capability areas of (i) detection and assessment; (ii) policy development, adaptation and implementation; (iii) health services; (iv) coordination and communication (within the PHEP system) and (v) emergency risk communication (with the public). The Table provides an overview of the number of competencies as well as knowledge and skill statements by capability area.

**Table:** Number of competency, knowledge and skill statements by capability area

Capability area	Number of		
	Competencies	Knowledge statements	Skill statements
Detection and assessment (incident recognition, risk characterisation, epidemiological investigation, surveillance and monitoring, laboratory analysis and environmental monitoring)	26	11	38
Policy development, adaptation and implementation for infection control and treatment guidance and for population-based disease control	17	27	21
Health services (preventive services; medical surge; management of medical countermeasures, supplies and equipment; and care for healthcare workers and emergency responders)	20	20	35
Coordination and communication within the PHEP system (crisis management and communication with healthcare providers, emergency management, public safety and other sectors, as well as other public health agencies at the global, European, national and subnational levels)	23	27	22
Emergency risk communication (the real-time exchange of information, advice and opinions between experts and officials, and people who face threats to their survival, health, economic or social well-being)	16	15	42

PHEP: public health emergency preparedness.

Source: Reproduced from [5].

To illustrate the results, the Box presents competencies, knowledge and skill statements related to incident recognition in the area of detection and assessment capability. These competencies are intended to apply to public health epidemiologists, national public health agency leaders and others who would be involved in leading the operational response to a public health emergency, such as the nominated ECDC National Focal Points for Preparedness and Response. The model includes three competency, four knowledge and four skill statements. The full set of competency, knowledge and skill statements for each capability in the logic model is available online [5].

Box **Competency, knowledge and skill statements for the incident-recognition capability**

**Table:** 

**Incident recognition: Identifying that a cross-border threat to health has arisen, either in one or more of the member states, or elsewhere in the world that could affect Europe**
*Workforce groups* • Comprised of public health epidemiologists, national public health agency leaders, NFPs for preparedness.
*Competencies* • Use event-based and indicator-based surveillance systems to detect health threats. • Know when case reports or clusters require further investigation, and how to initiate such investigations. • Evaluate the implications of national or international public health alerts for own country.
*Knowledge (K) and Skills (S)* • Know sources of information about potential public health threats from within own country (K). • Follow up on reported information about potential public health threats from within own country to assess its quality and validity (S). • Critically appraise information about potential public health threats from within own country (S). • Evaluate whether a potential public health threat from within own country is notifiable under the IHR (S). • Follow chains of communication to notify own countries’ IHR focal point about a potentially notifiable PHEIC (S). • Understand and critically appraise information from event-based and indicator-based surveillance systems (S). • Be familiar with laws on surveillance and reporting of communicable diseases at national, EU and global levels (K). • Be aware of sources of international public health alerts (K). • Understand and critically appraise international public health alerts to assess the implications for own country (K).

EU: European Union; IHR: international health regulations; NFP: national focal point; PHEIC: public health emergency of international concern.Reproduced and adapted from [5]. The complete list is available from [3].

## Conclusions

The proposed competency model was designed to support developers of PHEP training initiatives by ensuring that public health professionals are able to demonstrate the knowledge and skills needed for successful performance when preparing and responding to cross-border health threats. This research advances ECDC’s efforts to address cross-border threats to health by translating the capabilities in the PHEP logic model [2]—which apply at the system level—into competencies for individuals who work in the public health emergency preparedness system.

While an empirical verification of the proposed competency model was beyond the scope of our project, what distinguishes this model from previous efforts is the fact that competencies were derived from documented public health systems’ response gaps identified during the analysis of the response to specific public health incidents. Furthermore, a PHEP curriculum was developed and pilot tested during a 3.5-day, face-to-face training in conjunction with the Alma Mater Studiorum - Università di Bologna, in Bologna Italy in May 2018, leading to the creation of a toolkit for developers of PHEP trainings.

Knowledge and skills, of course, are necessary but not sufficient; national preparedness systems also need motivated practitioners, under dedicated leadership and strong public health systems as well as competency-based training. However, the competencies provide a common foundation upon which training programs can be based, thereby helping to realise the goals set out in the European Parliament and Council Decision 1082 on serious cross-border threats to health [1].
